# Nordihydroguaiaretic acid enhances the activities of aminoglycosides against methicillin- sensitive and resistant *Staphylococcus aureus in vitro* and *in vivo*

**DOI:** 10.3389/fmicb.2015.01195

**Published:** 2015-10-27

**Authors:** Edward Cunningham-Oakes, Odel Soren, Caroline Moussa, Getika Rathor, Yingjun Liu, Anthony Coates, Yanmin Hu

**Affiliations:** ^1^Institute for Infection and Immunity, St George’s, University of LondonLondon, UK; ^2^Centre for Biological Sciences, University of SouthamptonSouthampton, UK

**Keywords:** nordihydroguaiaretic acid, gentamicin, neomycin, tobramycin, *Staphylococcus aureus*, antibiotic combination

## Abstract

Infections caused by methicillin-sensitive *Staphylococcus aureus* (MSSA) and methicillin-resistant *S. aureus* (MRSA) are prevalent. MRSA infections are difficult to treat and there are no new classes of antibiotics produced to the market to treat infections caused by the resistant bacteria. Therefore, using antibiotic enhancers to rescue existing classes of antibiotics is an attractive strategy. Nordihydroguaiaretic acid (NDGA) is an antioxidant compound found in extracts from plant *Larrea Tridentata*. It exhibits antimicrobial activity and may target bacterial cell membrane. Combination efficacies of NDGA with many classes of antibiotics were examined by chequerboard method against 200 clinical isolates of MRSA and MSSA. NDGA in combination with gentamicin, neomycin, and tobramycin was examined by time-kill assays. The synergistic combinations of NDGA and aminoglycosides were tested *in vivo* using a murine skin infection model. Calculations of the fractional inhibitory concentration index (FICI) showed that NDGA when combined with gentamicin, neomycin, or tobramycin displayed synergistic activities in more than 97% of MSSA and MRSA, respectively. Time kill analysis demonstrated that NDGA significantly augmented the activities of these aminoglycosides against MRSA and MSSA *in vitro* and in murine skin infection model. The enhanced activity of NDGA resides on its ability to damage bacterial cell membrane leading to accumulation of the antibiotics inside bacterial cells. We demonstrated that NDGA strongly revived the therapeutic potencies of aminoglycosides *in vitro* and *in vivo*. This combinational strategy could contribute major clinical implications to treat antibiotic resistant bacterial infections.

## Introduction

There is no doubt that antimicrobial discovery and development experienced an era of prosperity in the not so distant past, known as the “golden era of antibiotics" (Aminov, 2010). However, this era of prosperity has come to an end due to rapid emergence of antibiotic resistance in bacteria and a dramatically reduced rate in discovery of new antibiotic classes (Sheridan, 2005). This matter is of great concern in the treatment of infections such as those caused by methicillin-resistant *Staphylococcus aureus* (MRSA). Methicillin-sensitive *S. aureus* (MSSA) and MRSA are major causes of life-threatening infections including surgical site infections, bacteraemia, pneumonia and catheter-associated infections (Fry and Barie, 2011; Magret et al., 2011), leading to significant morbidity and mortality. There is a very limited antimicrobial armamentarium to treat MRSA infections, of which vancomycin (a glycopeptide) and linezolid (an oxazolidinone antibiotic) are the major antibiotics. Worryingly, some cases of MRSA infections are also resistant to vancomycin (Liu and Chambers, 2003) and linezolid resistance has recently emerged (Gu et al., 2013). This combined with a significantly narrowed antibiotic pipeline (Butler et al., 2013) indicates that our ability to treat MRSA infections is rapidly diminishing (Lowy, 2003; Sheridan, 2005; Boucher et al., 2009). Therefore, using antibiotic enhancers (Kalan and Wright, 2011; Hu and Coates, 2012; Hu et al., 2015) as a means to revive existing classes of antibiotics provides an attractive strategy to combat the recent upsurge in antibiotic resistance. Aminoglycosides are not typically used to treat Gram-positive infections, but are known to exhibit a degree of anti-staphylococcal activity (Vakulenko and Mobashery, 2003). The potential that the anti-staphylococcal activities of aminoglycosides offer makes them particularly an attractive option. Additionally, revival of this class in treatment of MRSA would allow reduced vancomycin usage which ideally should only be used as a last resort.

**FIGURE 1:**
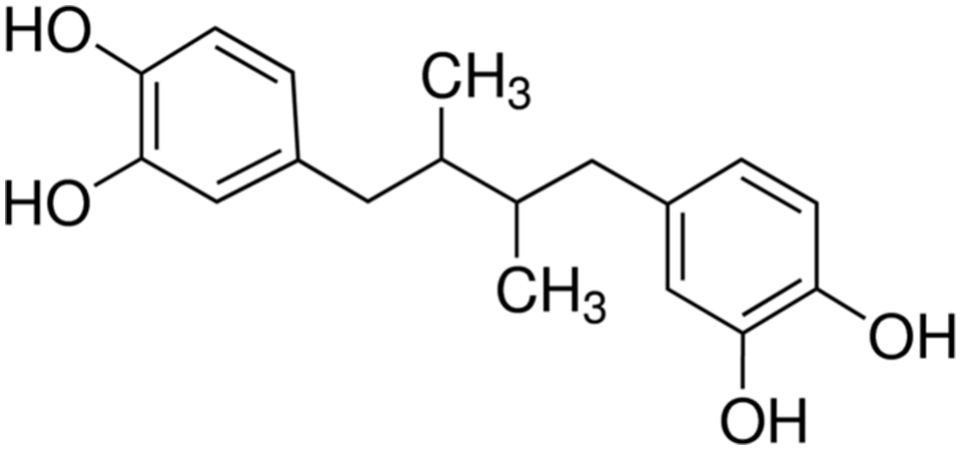
**Chemical structure of Nordihydroguaiaretic acid (NDGA)**.

Nordihydroguaiaretic acid (NDGA) is an antioxidant compound (**Figure 1**) found in extracts from *Larrea Tridentata*, a plant of great ethnobotanical importance. This antioxidant has a wide range of uses including tumor prevention, chemotherapy and treatment of the herpes simplex virus (Lu et al., 2010). It has been reported that NDGA showed anti-staphylococcal activity (Cowan, 1999; Gokbulut et al., 2013; Ooi et al., 2015), which may be due to the effect of NDGA on bacterial cell membrane (Ooi et al., 2015). Unfortunately, NDGA may possess potential adverse systemic effects such as non-competitive inhibition of sex hormones and nephrotoxicity (Lambert et al., 2002; Lu et al., 2010) at higher doses; the true nature of such effects is largely unknown (Lu et al., 2010). A recent study (Ooi et al., 2015) demonstrated that NDGA at low concentrations was able to increase the potency of gentamicin against *S. aureus* biofilm and suggested that the combination of NDGA with gentamicin could potentially be used to treat superficial staphylococcal infections. As NDGA might act on bacterial cell membrane (Ooi et al., 2015), potentially it could be used as an antibiotic enhancer (Hu and Coates, 2012) to boost the activities of other antibiotic such as aminoglycosides.

In this study, we retrospectively tested the *in vitro* and *in vivo* activities of NDGA in combination with many classes of antibiotics, but mainly focused on aminoglycosides using clinical isolates of MSSA and MRSA.

## Materials and Methods

### Bacterial Strains and Growth Conditions

Bacterial strains used were clinical isolates of MSSA (*n* = 100) and MRSA (*n* = 100) isolated from St George’s Hospital, London. These isolates were collected from blood cultures, tissue fluid, or routine screening on skin of the patients in the South West London area. Most of these strains were isolated from bacteremia. Some were isolated as organ or skin colonization. Genotyping of these strains were performed previously (Hu et al., 2015). The isolates were grown in nutrient broth (Oxoid) or on trypticase soy agar (Oxoid) plates.

### Susceptibility Tests of Antibiotics and NDGA

The minimum inhibitory concentration (MIC) was determined in 96-well plates using Iso*-*Sensitest broth (Oxoid) in accordance with the Clinical and Laboratory Standards Institute guidelines for broth microdilution MIC (Barry, 1999). Serial twofold dilutions of antibiotics were prepared in triplicate followed by addition of a standard bacterial suspension of 1–5 × 10^5^ colony forming unit (CFU)/mL. After 24 h incubation at 37°C, the optical density (OD) readings were measured using an ELx800 absorbance microplate reader (BioTek). The MIC was determined as the lowest concentration of an antibiotic with similar OD reading as the control (medium only). NDGA (purity at 97%) and the antibiotics used in the study were obtained from Sigma–Aldrich UK.

### Chequerboard Analysis to Determine Combination Effect of NDGA with Antibiotics

The chequerboard assay was used for the measurement of combination effects of NDGA with antibiotics. Combinations of two drugs were prepared using 96 well plates with drug concentrations starting from twofold higher than their MIC values, then serially diluted in a twofold manner. The two drugs were mixed in a 96 well plate followed by addition of a standard bacterial suspension of 1–5 × 10^5^ CFU/mL. After 24 h incubation at 37°C, the OD readings were determined using an EL × 800 absorbance microplate reader (BioTek). The combination effects were determined by calculating the fractional inhibitory concentration index (FICI) as follows**:** (MIC of drug A, tested in combination)/(MIC of drug A, tested alone) + (MIC of drug B, tested in combination)/(MIC of drug B, tested alone). The effects of the combination were defined as synergy if the FICI was ≤0.5, no interaction if the FICI was >0.5 but ≤4.0 and antagonism if the FICI was >4.0 (Odds, 2003).

### Time Kill Analysis of Aminoglycoside Combinations with NDGA against MSSA and MRSA

A range of different concentrations of NDGA and antibiotics singly or in combination were incubated with bacterial cultures containing 10^7^ CFU/mL of the test isolates. At 0, 1, 2, 4, 7, and 24 h of incubation, viability was determined by plating 100 μL of serial dilutions onto trypticase soya agar plates followed by incubation at 37°C for 24 h. The CFU was counted using aCOLyte colony counter (Synbiosis) and analyzed using the counter’s software. Viability was expressed at log CFU/mL.

### Measurement of ATP Release

The effect of NDGA on bacterial cytoplasmic membrane damage was measured by leakage of ATP according to the methods described previously (Johnston et al., 2003; Oliva et al., 2004; Ooi et al., 2009) using the CellTiter-Glo luminescent cell viability kit (Promega). *S. aureus* clinical strains were treated with different concentrations of NDGA and control antimicrobial agents for various time periods. Negative controls were included as CellTiter-Glo reagent with buffer and CellTiter-Glo reagent with bacterial cells without drug. The luminescence was measured using GloMax-Multi+ microplate reader (Promega).

### Mouse Superficial Skin Infection Model

The mouse superficial skin infection model was performed as described previously (Kugelberg et al., 2005; Hu and Coates, 2012) using female ICR mice (6–8 weeks old, Charles River). The mice were anesthetized by intraperitoneal injection of 200 μL of a 1:1:2 mixture of 100 mg/mL ketamine hydrochloride, 20 mg/mL xylazine, and sterile water. An area of 2 cm^2^ skin was tape-stripped using an autoclave tape after removal of the fur on the mouse back. The skin was stripped 10 times in succession. This procedure damaged the skin by removing the top dermal layers, which became red and shiny, but without observable bleeding. Buprenorphine was given at 0.2 mg/kg body weight during the anesthetic period and every 12 h to reduce pain. Bacterial infection was induced by the addition of 25 μL of log phase culture containing 10^7^ bacterial cells on the stripped skin. At 24 h after infection, treatment with the antibiotic formulation was initiated. At different time points after infection and treatment, three mice were sacrificed by cervical dislocation. The skin, approximately 2 cm^2^ was cut and added to 2 mL tubes which contained 1 mL water and glass beads (1 mm). The skin was homogenized using a SpeedMill Plus^TM^ (AnalytikJena) homogenizer for 1 min. Antibiotic remaining on the skin was removed by washing three times with water. CFU counts were performed on serial dilutions of the homogenates.

All animal experiments were performed according to the Animals Scientific Procedures Act, 1986 (an Act of the Parliament of the United Kingdom 1986 c. 14; Home Office Project licence Number 70/7077) with approval from St George’s, University of London ethics committee.

### Statistical Analysis

The significance of differences between experimental groups was determined by Student’s *t*-test. *P*-values <0.05 were considered significant.

## Results

### Chequerboard Analysis of NDGA-Antibiotic Combination Activities

The activities of antibiotics such as amoxicillin, penicillin, gentamicin, neomycin, tobramycin, clarithromycin, and erythromycin in combination with NDGA were determined using the chequerboard method against the 100 MSSA and 100 MRSA isolates. Synergistic activities were only observed when NDGA was combined with gentamicin, neomycin, and tobramycin for both MSSA and MRSA. As seen in **Table 1**, MICs for gentamicin, neomycin, and tobramycin for MSSA ranges from 0.125 to 1 mg/L, 0.5 to 1 mg/L, and 0.125 to 1 mg/L, respectively, showing all strains being susceptible to the aminoglycosides (break point for gentamicin and tobramycin is 1 mg/L; EUCAST, 2013). However, for the MRSA strains, although 50% of the strains were susceptible, some strains were highly resistant to the three antibiotics. There are 18 gentamicin resistant, 25 neomycin resistant, and 27 tobramycin resistant MRSA (Supplementary Data Sheet S1). NDGA showed high MIC value against both MSSA and MRSA (**Table 1**).

**Table 1:** Minimal inhibitory concentration (MIC) of gentamicin, neomycin, tobramycin, and Nordihydroguaiaretic acid (NDGA) against clinical isolates of methicillin-sensitive *Staphylococcus aureus* (MSSA) and methicillin-resistant *S. aureus* (MRSA).

			MIC^a^ (mg/L)	
		
Bacterial strains	Agents^b^	MIC range	MIC_50_	MIC_90_
MSSA^c^	Gentamicin	0.125–1	0.25	0.5
	Neomycin	0.5–1	0.5	1
	Tobramycin	0.125–1	0.25	0.5
	NDGA	16–128	32	32
MRSA^d^	Gentamicin	0.03–512	0.125	16
	Neomycin	0.06–512	0.5	32
	Tobramycin	0.03–512	0.125	64
	NDGA	8–64	32	64


*^a^MIC_50_ and MIC_90_ 50% and 90% indicate that MIC values at which 50 and 90% of the isolates were inhibited, respectively. ^b^Break point for gentamicin and tobramycin is 1 mg/L (EUCAST, 2013). Break point for neomycin and NDGA are not available. ^c^*n* = 100; ^d^*n* = 100.*

**Table 2:** Combination activities of NDGA with gentamicin, neomycin, and tobramycin against MSSA and MRSA.

			Total no. (%) of strains detected when NDGA combined with		
		
Strains	Combination activity	FICI	Gentamicin	Neomycin	Tobramycin
MSSA	Synergy	<0.5	99(99%)	100 (100%)	100 (100%)
	No interaction	0.56–4	1 (1%)	0	0
	Antagonism	>4	0	0	0
MRSA	Synergy	<0.5	98(98%)	100 (100%)	97 (97%)
	No interaction	0.56–4	2 (2%)	0	3 (3%)
	Antagonism	>4	0	0	0


Combination of gentamicin, neomycin, and tobramycin with NDGA (**Table 2**) displayed synergistic interaction against 99, 100, and 100% of MSSA and 98, 100, and 97% of MRSA isolates, respectively. Additionally, the MICs of all three aminoglycosides were significantly reduced from 2- to 128-fold against MSSA (Supplementary Data Sheet S2) and 2- to 256-fold against MRSA (Supplementary Data Sheet S1) when used in combination with NDGA, significantly, for the antibiotic resistant MRSA isolates (Supplementary Data Sheet S1).

### Time Kill Analysis of Combinations against MRSA and MSSA

**FIGURE 2:**
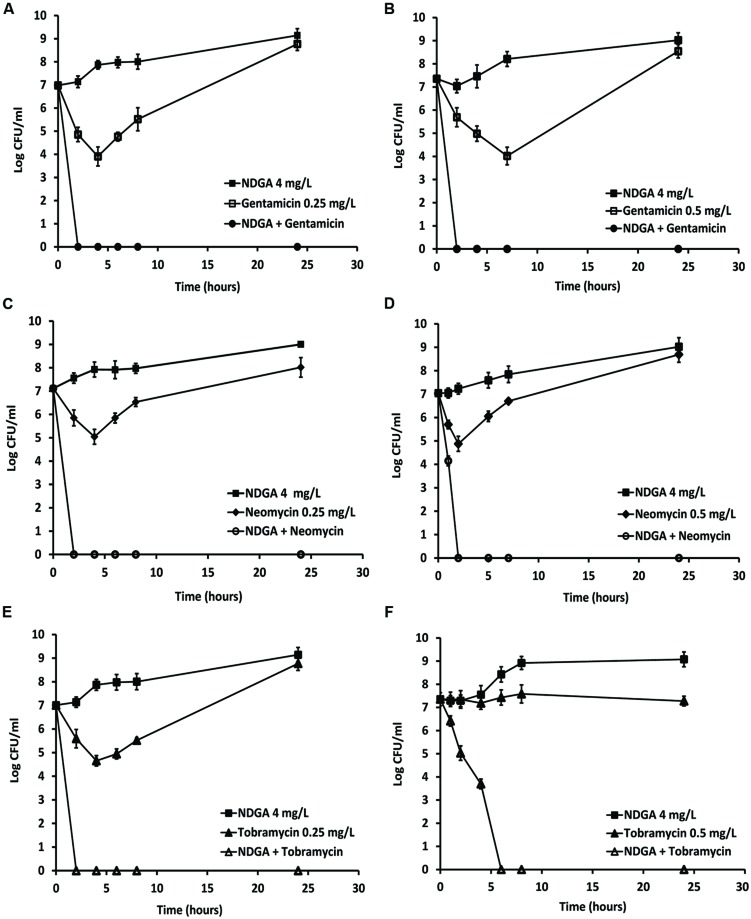
**Time-kill analysis showing the effects of NDGA in combination with gentamicin, neomycin, and tobramycin against log phase methicillin-sensitive *Staphylococcus aureus* (MSSA) and methicillin-resistant *S. aureus* (MRSA).** NDGA and the antibiotics alone or each antibiotic combined with NDGA were added to the log-phase cultures and colony forming unit (CFU) counts were carried out at different time points. Concentrations of NDGA was 4 mg/L. The three aminoglycoside concentrations were 0.25 mg/L for MSSA and 0.5 mg/L for MRSA. Combination of NDGA with gentamicin against MSSA **(A)** and MRSA **(B)**. Combination of NDGA with neomycin against MRSA **(C)** and MRSA **(D)**. Combination of NDGA with tobramycin against MSSA **(E)** and MRSA **(F)**. Results shown are mean of two independent experiments.

The synergistic activity of NDGA in combination with gentamicin, neomycin, and tobramycin was examined in seven strains of both MRSA and MSSA. The range of concentrations for each drug was determined according to the results obtained from chequerboard which displayed an FIC index <0.25. The combinations with the lowest concentration of NDGA (4 mg/L) which stimulated the most effective synergistic activity are shown. Dose dependent effects of NDGA enhancement for gentamicin, neomycin and tobramycin against MSSA and MRSA are shown in Supplementary Data Sheet 3. As seen in **Figure 2**, NDGA at 4 mg/L showed no effect against both MSSA and MRSA strains. Gentamicin at a concentration of 0.25 (MSSA) and 0.5 (MRSA) mg/L displayed initial bacterial kill, but regrowth was seen after 4 h (**Figure 2A**) of drug exposure for MSSA and 7 h (**Figure 2B**) for MRSA. However, gentamicin and NDGA combination reduced the initial inoculum from 10^7^ CFU/ml to zero at one hour for both strains (**Figures 2A,B**). As shown in **Figures 2C,D**, bactericidal effects of neomycin at concentrations of 0.25 (MSSA) and 0.5 (MRSA) mg/L were observed initially but diminished by bacterial regrowth. However, in combination with 4 mg/L of NDGA, rapid kill (100% reduction) was seen at 1 h for MSSA and 2 h for MRSA. Tobramycin at 0.25 mg/L was bactericidal against the MSSA at 4 h of drug treatment followed by regrowth (**Figure 2E**) and 0.5 mg/L inhibited the MRSA growth (**Figure 2F**). In combination with NDGA, complete kill was seen at 2 h for MSSA and 6 h for MRSA. There were no regrowth for the combination of NDGA with all three aminoglycosides at 24 and 48 h of (Data not shown) incubation. The regrowth that occurred following single drug treatment may be attributable to drug degradation or as a result of microorganism adaptation (Chan and Zabransky, 1987; Aeschlimann et al., 1999). The similar combination profiles of NDGA and the aminoglycosides were confirmed in different strains of MSSA and MRSA (Data not shown).

### NDGA Boosted the Effects of Gentamicin, Neomycin, and Tobramycin against MRSA and MSSA in Mouse Skin Infection Model

**FIGURE 3:**
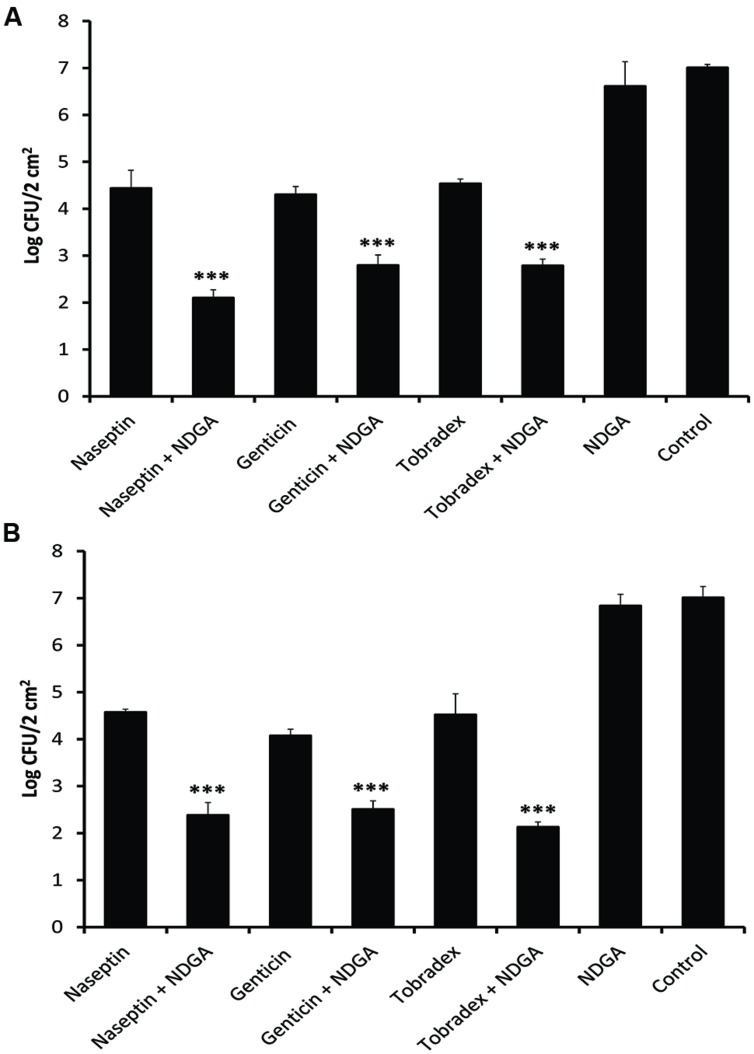
**Effect of NDGA boosting the effects of currently marketed formulations containing gentamicin, neomycin, and tobramycin against MSSA and MRSA in a murine skin bacterial infection model.** Viability of the bacteria was determined after 24 h of treatment. Treatment with NDGA, Genticin, Naseptin, and Tobradex singly or in combination against MSSA **(A)** and MRSA **(B)**. The data has been repeated twice. ^∗∗∗^*P* < 0.001.

We investigated whether NDGA would synergise and improve the therapeutic effects of topical aminoglycoside formulations currently on the market. Naseptin^®^(0.5% neomycin sulfate and 0.1% chlorhexidine hydrochloride, Alliance), Genticin^®^(0.3% gentamicin, Amdipharm), or Tobradex^®^(tobramycin 0.3% and dexamethasone 0.1%, Alcon) alone and in combination with NDGA (32 mg/L) were tested against one MRSA and one MSSA on mouse skin. Both test strains were isolated from bacteremia with a NDGA MIC of 32 mg/L and gentamicin, neomycin, and tobramycin MIC of 1, 0.5, and 1 mg/L; respectively. These strains showed FIC indices <0.5 and significant synergistic activities in time kill curve for each of the drug combinations. After a bacterial infection was established at 24 h after inoculation, 45 μL of either Genticin^®^, Naseptin^®^, Tobradex^®^, NDGA, combination of NDGA with the formulations and placebo were applied to the infected area. Bacterial CFU counts were then taken after 24 h of treatment. As shown in **Figure 3**, NDGA showed no effect as a single agent (*n* = 3) whilst Genticin^®^, Naseptin^®^, and Tobradex^®^ all have similar effects (*n* = 3), causing a reduction of 2.4–2.9 log in CFU counts against both MSSA (**Figure 3A**) and MRSA (**Figure 3B**). However, the effects of each formulation in combination with NDGA (*n* = 3) were significantly augmented (*P* < 0.001), leading to CFU count reductions of 4.2–4.9 logs compared to the placebo control in both MSSA and MRSA infections.

### Determining the Effect of NDGA on Bacterial Cytoplasmic Membrane Damage

**FIGURE 4:**
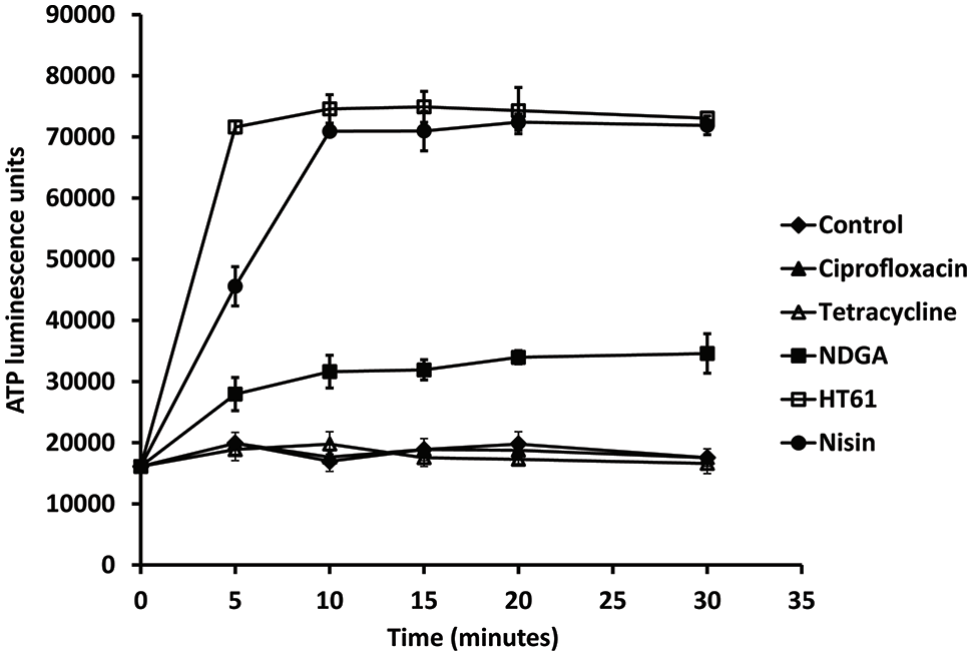
**Effect of NDGA and other antimicrobial agents on leakage of ATP against a clinical isolate of MSSA.** The bacterial strain was resuspended in HEPES buffer supplemented with glucose and treatment with ciprofloxacin, tetracycline, NDGA, HT61, and nisin. The experiments have been repeated twice with reproducible results.

Bacterial membrane damage was examined by ATP leakage after exposure of the *S. aureus* strain to NDGA and other antimicrobial agents. As shown in **Figure 4**, ciprofloxacin and tetracycline (4× MIC) resulted in no ATP leakage which was similar to previous finding (Oliva et al., 2004). The cell membrane damage agents HT61 (1× MIC) and nisin (4× MIC) permeabilized the cell membrane leading to leakage of significant amounts of ATP. However, NDGA (4× MIC) exposure produced amounts of ATP which was more than 40% of the control value over the period tested indicating that NDGA was a cytoplasmic membrane damage agent (Hilliard et al., 1999).

## Discussion

In this study, we showed that NDGA boosted the antibacterial efficacies of aminoglycosides, namely gentamicin, neomycin, and tobramycin, against MSSA and MRSA both *in vitro* and in a mouse infection model. The use of NDGA as an antibiotic enhancer may contribute to the production of novel therapeutic topical regimens for the treatment of antibiotic-resistant *S. aureus* infections.

Although aminoglycosides are highly effective broad spectrum antibiotics with anti-staphylococci activities (Vakulenko and Mobashery, 2003), they exhibit a wide range of clinically significant side-effects. These include nephrotoxicity or ototoxicity affecting the vestibulo-cochlear system (Selimoglu, 2007). The potential for irreversible nephrotoxicity limits neomycin to topical or oral administration (Edson and Keys, 1983). Overall, toxic side-effects significantly curtail the therapeutic usage of aminoglycosides in the status quo (Quiros et al., 2011; Mazurek et al., 2012).

In this study, NDGA alone showed no significant anti-staphylococcal activities against MSSA and MRSA showing MIC values between 16 and 128 mg/L, which confirmed previous findings (Ooi et al., 2015). When NDGA was used in combination with gentamicin, neomycin, and tobramycin, significant synergistic activity was observed against more than 97% of the test isolates. The synergistic interactions observed here were confirmed by time-kill assays, which has been repeatedly shown to be superior to chequerboard analysis (Hu and Coates, 2012; Hu et al., 2015) as it measures dynamically the antimicrobial activities of the combination over time. Our time kill studies showed that gentamicin at 2 to 4 mg/L, neomycin at 2 to 8 mg/L, and tobramycin 2 to 4 mg/L, led to complete eradication of log phase MSSA or MRSA within 1 to 4 h (data not shown). Lower concentrations of 0.25 to 0.5 mg/L of all three aminoglycosides showed initial bactericidal activity followed by regrowth (**Figure 2**). Importantly, after addition of 4 mg/L of NDGA, rapid bactericidal activities (100% kill) were seen with low doses of the aminoglycosides (**Figure 2**) which gave the equivalent potency of the antibiotic used singly to achieve a complete kill. Furthermore, the culture remained free of bacteria even after 48 h of incubation (data not shown) indicating that all the bacteria were eliminated. The enhancement of aminoglycoside bactericidal activities by NDGA is important as therapeutic effects can be achieved with lower doses of aminoglycoside and this dose reduction would significantly reduce the potential for toxic side effects. Aminoglycoside enhancer has been shown previously (Mitchell et al., 2012) to increase the drug potency against both antibiotic-susceptible and antibiotic-resistant *S. aureus*. Bacterial strains used in this study were clinical isolates randomly selected from patients with a range of infections, the majority of which were associated with bacteraemia (Hu et al., 2015). These isolates were genotypically diverse and included representatives of the dominant lineages of *S. aureus* recovered from humans (Hu et al., 2015). This strongly supports the notion that NDGA-aminoglycoside combinations are effective in a genotypically diverse population of *S. aureus*.

The therapeutic potential of NDGA as an aminoglycoside enhancer was demonstrated on the treatment of mouse MSSA and MRSA skin infections. We clearly showed that combinations of NDGA with current marketed formulations, Genticin (gentamicin), Naseptin (neomycin), Tobradex (tobramycin), significantly boosted the efficacies of the formulations and reduced around 2 logs more of the bacterial loads on the skin. These enhanced effects are particularly important as the fast action of the combination is likely to shorten the treatment duration. Since long-term administration of aminoglycosides render bacteria susceptible to the development of antibiotic resistance (Buckwold et al., 1979), the potential to shorten antibiotic therapy duration and still achieve the same degree of therapeutic activities can reduce antibiotic resistance emergence which may improve clinical outcomes (Magana et al., 2015).

Increasing cell permeability is one of the major modes of action for antibiotic enhancers (Hu and Coates, 2012; Hu et al., 2015; Magana et al., 2015). Compounds which target the cell wall or cell membrane have been shown to boost the therapeutic activities of antibiotics (Giacometti et al., 2000; Anantharaman et al., 2010; Hu and Coates, 2012; Hu et al., 2015). It is well established that membrane potential is a major factor determining the degree of aminoglycoside uptake (Mates et al., 1982). We showed that NDGA exposure led to bacterial ATP leakage indicated that NDGA damaged *S. aureus* cell membrane which is in agreement with the finding reported previously (Ooi et al., 2015). Aminoglycosides inhibit bacterial protein synthesis and their bactericidal activities are concentration dependent (Hancock, 1981). Therefore, increased bactericidal activities are associated with increased bacterial intracellular concentration of the antibiotics. The effect of the increased membrane permeability induced by NDGA resulted in accumulation of the antibiotics inside bacterial cells leading to increased levels of the antibiotics intracellularly, thus contributed enhanced bacterial kill. Similar studies showed that cell membrane damage agent enhanced aminoglycoside activities against *S. aureus* (Hu and Coates, 2012).

## Conclusion

This study showed that NDGA in combination with aminoglycosides can successfully remove both MSSA and MRSA *in vitro* and significantly reduced the bacterial burden *in vivo*. This combination therapy may allow a lower dose of aminoglycosides to be used while maintaining therapeutic potency. The ability of NDGA to rejuvenate the efficacy of the antibiotics resides in its ability to permeabilize bacterial cytoplasmic membrane with a potential to allow accumulations of the combined antibiotics in the target site. Our groundwork has provided early preclinical assessments of NDGA combination with aminoglycosides and lays the foundation for further validation for clinical usage.

## Author Contributions

YH: conceived and designed the experiments; EC-O, OS, CM, GR, YL, YH: performed the experiments; YH, EC-O: analyzed the data; YH, AC: contributed reagents/materials/analysis tools; YH, EC-O: wrote the paper.

## Conflict of Interest Statement

Yanmin Hu and Anthony Coates are shareholders in Helperby Therapeutics Limited. The authors declare that the research was conducted in the absence of any commercial or financial relationships that could be construed as a potential conflict of interest.
